# Plasma Metabolomics Reveals Dysregulated Metabolic Signatures in HIV-Associated Immune Reconstitution Inflammatory Syndrome

**DOI:** 10.3389/fimmu.2021.693074

**Published:** 2021-06-15

**Authors:** Luxin Pei, Kiyoshi F. Fukutani, Rafael Tibúrcio, Adam Rupert, Eric W. Dahlstrom, Frances Galindo, Elizabeth Laidlaw, Andrea Lisco, Maura Manion, Bruno B. Andrade, Irini Sereti

**Affiliations:** ^1^ Laboratory of Immunoregulation, National Institute of Allergy and Infectious Diseases (NIAID), National Institutes of Health (NIH), Bethesda, MD, United States; ^2^ Department of Biology, Johns Hopkins University, Baltimore, MD, United States; ^3^ Multinational Organization Network Sponsoring Translational and Epidemiological Research (MONSTER) Initiative, Salvador, Brazil; ^4^ Laboratory of Inflammation and Biomarkers, Gonçalo Moniz Institute, Oswaldo Cruz Foundation, Salvador, Brazil; ^5^ Curso de Medicina, Centro Universitário Faculdade de Tecnologia e Ciências (UniFTC), Salvador, Brazil; ^6^ Faculdade de Medicina, Universidade Federal da Bahia, Salvador, Brazil; ^7^ Leidos Biomedical Research Inc., Frederick National Laboratory for Cancer Research, Frederick, MD, United States; ^8^ Rocky Mountain Laboratories, National Institute of Allergy and Infectious Diseases (NIAID), National Institutes of Health (NIH), Hamilton, MT, United States; ^9^ Wellcome Centre for Infectious Disease Research in Africa, Institute of Infectious Disease and Molecular Medicine, University of Cape Town, Cape Town, South Africa; ^10^ Division of Infectious Diseases, Department of Medicine, Vanderbilt University School of Medicine, Nashville, TN, United States

**Keywords:** immune reconstitution inflammatory syndrome (IRIS), cell metabolism, metabolomics, immune activation, HIV

## Abstract

Immune reconstitution inflammatory syndrome (IRIS) is an inflammatory complication associated with an underlying opportunistic infection that can be observed in HIV-infected individuals shortly after the initiation of antiretroviral therapy, despite successful suppression of HIV viral load and CD4^+^ T cell recovery. Better understanding of IRIS pathogenesis would allow for targeted prevention and therapeutic approaches. In this study, we sought to evaluate the metabolic perturbations in IRIS across longitudinal time points using an unbiased plasma metabolomics approach as well as integrated analyses to include plasma inflammatory biomarker profile and whole blood transcriptome. We found that many lipid and amino acid metabolites differentiated IRIS from non-IRIS conditions prior to antiretroviral therapy and during the IRIS event, implicating the association between oxidative stress, tryptophan pathway, and lipid mediated signaling and the development of IRIS. Lipid and amino acid metabolic pathways also significantly correlated with inflammatory biomarkers such as IL-12p70 and IL-8 at the IRIS event, indicating the role of cellular metabolism on cell type specific immune activation during the IRIS episode and in turn the impact of immune activation on cellular metabolism. In conclusion, we defined the metabolic profile of IRIS and revealed that perturbations in metabolism may predispose HIV-infected individuals to IRIS development and contribute to the inflammatory manifestations during the IRIS event. Furthermore, our findings expanded our current understanding IRIS pathogenesis and highlighted the significance of lipid and amino acid metabolism in inflammatory complications.

## Introduction

Antiretroviral therapy (ART) effectively controls HIV viral replication and leads to the restoration of immune function, which has greatly improved the life expectancy of people living with HIV (PWH). Growing evidence suggests that a fraction of HIV-infected patients, however, can still develop severe inflammatory complications and experience clinical deterioration within the first few weeks following the initiation of ART despite successful suppression of HIV viral load and recovery of CD4^+^ T cells (1). This condition, termed immune reconstitution inflammatory syndrome (IRIS), presents with clinical manifestations such as worsening lymphadenopathy, fever, malaise, and worsening pulmonary infiltrates even with microbiologic control of the underlying co-infection. Notably, mycobacterial co-infections, such as *Mycobacterium tuberculosis* (TB) and *Mycobacterium avium* complex (MAC), are frequently associated with IRIS that can lead to higher morbidity and mortality rates (2–5). The incidence of IRIS can vary from <5% to as high as 50% and is dependent on several risk factors including severe lymphopenia prior to starting ART as well as disseminated infection with high antigen load (6). Current management for IRIS involves clinical observation, drainage of inflammatory collections, use of non-steroid anti-inflammatory drugs (NSAIDs), or use of corticosteroids for either prevention or treatment in high-risk TB patients (5, 7, 8).

A comprehensive understanding of IRIS is evolving, and it is now well appreciated that IRIS pathogenesis is characterized by dysregulated host innate and adaptive immune responses to the underlying co-infection. More specifically, IRIS is associated with hyperactivation of polyfunctional antigen-specific CD4^+^ T cells resulting in exaggerated production of pro-inflammatory cytokines such as TNF and IFN-γ (3, 9–14). Additionally, patients who develop IRIS display monocyte activation with increased production of pro-inflammatory cytokines along with altered gene expression profile both prior to ART initiation and during the IRIS event (6, 15). Elevated levels of soluble plasma biomarkers, cytokines, and chemokines associated with both adaptive and innate immune activation have also been described in IRIS including IL-6, IL-8, granulocyte-macrophage colony-stimulating factor (GM-CSF), sCD14 and the afore mentioned TNF and IFN-γ (3, 6, 16–18). IL-6, IL-8, GM-CSF, and sCD14 are of innate immune origins reflecting pathogen activation of monocytes and macrophages (6, 16, 18). Chemokine IL-8 is also responsible for neutrophil recruitment to sites of inflammation (16). The increased levels of IFN-γ and TNF indicate a T-helper 1 bias in IRIS T cell responses. Furthermore, the role of pro-inflammatory cytokines in mediating IRIS pathogenesis is elucidated through blockade or ablation of IFN-γ, TNF, and IL-6 in a MAC-IRIS murine model as well as in IRIS patients who are refractory to steroid treatment (10, 11, 19). Such robust systemic inflammation observed in IRIS may be reflected in substantial immunometabolic shifts (5). Indeed, IRIS was recently linked with higher metabolic activity monitored by nuclear imaging technique ^18^F-fluorodeoxyglucose positron emission tomography (FDG-PET). FDG-PET results showed higher total glycolytic activity and standardized uptake values in IRIS patients, which was also supported by increased expression of glucose transporter 1 (GLUT-1) on both CD4^+^ T cells and CD14^+^ monocytes (20).

A large number of studies underscores the importance of intracellular metabolism for the maintenance of both T cell and monocyte functions, since immune cells must cope with different catabolic and anabolic demands in response to antigen activation and other inflammatory stimuli. In particular, metabolic reprogramming characterized by glycolytic shift and increased mitochondria function greatly contributes to T cell effector function and macrophage activation (21–28). Therefore, the identification of immunometabolic requirements as well as dysfunctional metabolic pathways may further unravel the nuances surrounding IRIS pathogenesis.

The application of metabolomics offers an untargeted global approach for the identification of distinct metabolic signatures. Metabolomics studies have demonstrated that immune activation associated with mycobacterial infections or autoimmune conditions substantially change the metabolic state of the immune system, which, in turn, could also affect the host response to the pathogen (29–34). To comprehend how metabolic disturbances contribute to IRIS development and progression, we devised an unbiased plasma metabolomics approach to identify altered metabolite composition at longitudinal time points in PWH who developed IRIS compared to those who did not upon ART commencement. Our findings suggested alterations of the metabolic profile in IRIS patients mainly occurred before the initiation of ART and during the IRIS event with perturbed lipid and amino acid metabolism that further correlated with plasma inflammatory biomarkers.

## Materials and Methods

### Study Design and Patient Cohort

HIV infected ART-naïve patients with CD4^+^ T cell count <100 cells/μL were enrolled in a prospective observational study at the National Institutes of Health [PET Imaging and lymph node assessment of IRIS in persons with AIDS (PANDORA) NCT02147405]. The study was approved by the ethics committee and all participants signed informed consent prior to any study procedures. ART was started within 2 weeks of study enrollment following standard treatment guidelines. IRIS was diagnosed based on the AIDS Clinical Trials Group IRIS definition criteria including CD4^+^ T-cell count increased by ≥50 cells/µl or >two-fold from pre-ART levels and/or HIV plasma RNA reduced by >0.5 log_10_ copies/mL, and patient experienced signs and symptoms of inflammatory conditions attributed to a specific pathogen or condition that were not consistent with the development of a new infection, predicted clinical course of a pre-existing infection, or side effects of ART (4).

### Metabolomics

Cryopreserved plasma samples from 13 IRIS and 17 non-IRIS HIV-infected patients at the pre-ART, 1-2 months, and 12 months after ART initiation time points were collected. Non-targeted metabolomics analysis was performed at Metabolon, Inc using previously published method (35). Briefly, plasma samples underwent methanol extraction, and the resulting extract was used for metabolite analysis by ultra-high-performance liquid chromatography/tandem mass spectrometry in both positive and negative ion modes along with gas chromatography/mass spectrometry to maximize compound detection and accuracy. Metabolites were then identified by comparing the spectral signature of sample metabolites to a reference library at Metabolon, Inc. Spectral peaks were used for metabolite identification by a proprietary visualization and interpretation software. Area under the curve for the spectral peaks was used for metabolite quantification. Raw data generated from peak quantification were then normalized to correct for variation in multi-day experiments, where each compound was normalized to a median equal to one. The raw values after normalization are included in **Supplementary Table 1**.

### Plasma Biomarker Measurements

Concentrations of inflammatory biomarkers, including soluble PD-1, soluble CD14, IL-6r, MCP1, GMCSF, TNF, IL-8, IL-6, IL-2, IL-1β, IL-12p70, IL-10, IFN-γ, and MPO, were measured in cryopreserved plasma samples at the pre-ART and month 1 time points using a custom Meso Scale Discovery electrochemiluminescence multiarray kit following manufacturer recommendations. D-dimer was measured by an enzyme-linked fluorescent assay on a VIDAS instrument (bioMérieux, Durham, North Carolina).

### Statistical Methods

Comparisons of patients’ baseline characteristics between the IRIS, non-IRIS, and mycobacterial-IRIS groups were performed using the nonparametric Mann-Whitney *U* test. Frequencies of female sex and race were assessed using Chi-square test. BMI at the pre-ART, month 1, and month 12 time points were compared using the Wilcoxon signed rank test.

Differentially expressed metabolites (DEMs) were determined by multiple t-tests in a log_2_ transformed matrix comparing individual metabolite levels of IRIS and non-IRIS patients. FDR correction was not made for the identification of DEMs given the exploratory nature of the study. Venn diagram was used to visualize unique and shared differentially expressed metabolites. Hierarchical cluster analysis was performed using the Ward’s method (with 100X bootstrap), and principal component analysis (PCA) was performed using JMP Statistical Discovery PRO (Version 13). Decision trees were employed to identify a minimal set of markers allowing separation between IRIS from non-IRIS, and mycobacterial IRIS and other types of IRIS using J48 algorithm implemented in the WEKA program (Waikato Environment for Knowledge Analysis, version 3.6.11, University of Waikato, New Zealand) (36). In order to estimate the classification accuracy of decision tree models, we performed the sensitivity and specificity measurement using the receiver–operating characteristic curve (ROC) in JMP Statistical Discovery PRO (Version 13).

Co-expression module analysis for metabolic pathways was executed using the CEMiTool package (37). This package computes and identifies modules based on co-expressed/regulated pathways that were altered in a specific sample group. Module pathways were annotated based on reference library provided by Metabolon Inc. The file was in a *.gmt set and the enrichment values of each patient was used in correlation plots. The correlation profiles between metabolic pathways and plasma biomarkers at different time points were examined using Spearman correlation matrices. Only statistically significant correlations (*p*-values < 0.05 with r values above 0.7 and below -0.7) were included in the network visualization. Circos plots were used to illustrate the networks as previously reported (38).

### RNA Extraction and Library Preparation

Whole blood samples from the pre-ART, month 1, and month 12 post-ART longitudinal time points were collected and sequenced in 2 batches indicated in **Supplementary Table 1**. All whole blood samples were extracted using the PAXgene 96 Blood RNA Kit (Qiagen, Valencia, CA) following manufacturer’s instructions. RNA quality was assessed using 2100 Bioanalyzer RNA Pico 6000 kit (Agilent Technologies, Santa Clara, CA). Following total RNA extraction, each sample was subjected to purification steps using Agencourt RNAClean XP beads and Globin Removal Mix and instructions provided in the TruSeq Stranded Total RNA Sample Preparation Guide (Illumina, Guide, Part# 15021048, Rev E).

For the first set of samples, the TruSeq Stranded Total RNA Sample Preparation Kit was used to prepare sequencing libraries exactly as specified in the manufacturer’s recommended procedure followed by the usage of the RNA Adapter Plate for dual indexing (Illumina, Guide, Part# 15021048, Rev. E). The final libraries were assessed on the 2100 Bioanalyzer using the DNA 1000 chip (Agilent Technologies) and quantified using the Kapa Quantification Kit for Illumina Sequencing (Kapa Biosystems, Boston, MA) on the CFX384 Real-Time PCR Detection System (Bio-Rad Laboratories, Inc, Hercules, CA). Libraries were then prepared for clustering to the flow cell. On-board cluster generation and paired-end 76 base pair sequencing were completed on the NextSeq550 (Ilumina, Inc, San Diego, CA) using a High Output 150 cycle reagent kit. Three more NextSeq runs were completed to increase the mapping density. For the second set of samples, the TruSeq Stranded mRNA Sample Preparation Kit was used to prepare sequencing libraries exactly as specified in the manufacturer’s recommended procedure followed by the usage of the RNA Adapter Plate for dual indexing (Illumina, Guide, Part# 15031047, Rev. E). The final libraries were assessed on the 2100 Bioanalyzer using the DNA 1000 chip (Agilent Technologies). The fragment size distribution of the libraries was within the manufacturer’s specifications. TruSeq libraries were quantified on the CFX96 Touch real-time PCR instrument (BioRad, Hercules, CA) using the Kapa Library Quant Universal qPCR mix and kit instructions (Kapa Biosystems, Wilmington, MA). All samples were individually sized and normalized to a 2nM concentration. Samples were combined in equimolar ratios to create a single pool, titrated to 9pM, and sequenced as 2 x 93 bp reads on the HiSeq 2500 instrument using the HiSeq Rapid SBS 200 cycle kit, according to the manufacturer’s recommended procedure (Illumina, San Diego, CA). A total of four Rapid runs were performed to increase the mapping coverage. Targets from the two batches were mapped and identified by ENSG. Batch correction was performed using ComBat-seq (https://github.com/zhangyuqing/ComBat-seq) from the sva package to minimize experimental variance prior to subsequent analysis.

### Multi-Omics Factor Analysis (MOFA)

Multi-omics factor analysis (MOFA) is a computation method that provides the characterization and visualization of multi-layered biological processes to analyze the heterogeneity between IRIS and non-IRIS conditions (39–42). In total, 8 IRIS and 12 non-IRIS patients at the pre-ART and month 1 time points with paired metabolome, transcriptome, and concentration of plasma biomarker measurements were included in the analysis. The MOFA model was employed to integrate the omics data with a series of parameters including only paired samples in all datasets, selection of factors with the removal of zero variance, and factor tolerance to establish an exploratory model (tolerance = 1). The mixture of variables with similar variance was represented as latent factors based on an unsupervised factor analysis. The MOFA model is able to automatically determine Gaussian and Poisson distributions. All pipelines and analysis are available in http://www.bioconductor.org/packages/devel/bioc/html/MOFA.html.

## Results

### Patient Characteristics

Thirty HIV-infected ART naïve patients (22 males, 8 females) were identified in the study cohort with a median age of 37 years [interquartile range (IQR), 34-41]. Baseline demographic and clinical characteristics are shown in **Table 1**. The sex proportion was comparable and not significantly different between IRIS (female 23%) and non-IRIS (female 29%) groups (*p-*value=0.697). Prior to ART initiation, the median CD4^+^ T cell count of all patients was 19 cells/μL (IQR, 9-42), and plasma HIV-RNA was 5.3 log_10_ copies/mL (IQR, 4.9-5.8). Thirteen patients were diagnosed with IRIS as previously described (20) with a median time between ART initiation and onset of IRIS of 30 days (IQR, 27.5-36). There were no statistically significant differences in demographics and clinical characteristics between the IRIS and non-IRIS patients. The detailed descriptions of IRIS types, co-infections, IRIS onset date, and treatment are listed in **Supplementary Table 1**. Among the 13 IRIS patients, seven were diagnosed with mycobacterial IRIS (TB or MAC). Other types of IRIS included cryptococcal, Kaposi’s sarcoma, and progressive multifocal leukoencephalopathy IRIS (**Supplementary Table 1**). All patients had significant CD4^+^ T cell count recovery as well as HIV-RNA viral load suppression after the initiation of ART (**Supplementary Figures 1A, B**). A significant increase in BMI was also observed in all patients after ART at the month 1 and month 12 time points (p<0.001) (**Supplementary Figure 1C**).

**Table 1:** Baseline Demographic and Clinical Characteristics of Study Participants.

	All Patients (n = 30)	Non-IRIS (n = 17)	IRIS (n = 13)	*P*-value IRIS *vs*. Non-IRIS
Age, years median (IQR)	37 (34-41)	36 (35-41)	37 (33-43)	0.812
Female sex, No. (%)	8 (27)	5 (29)	3 (23)	0.697
Race,				
White	1	0	1	
African American	14	8	6	
Hispanic	14	8	6	
Asian	1	1	0	
BMI, kg/m^2^, median (IQR)	22.7 (19.4-24.6)	22.7 (18.6-24.8)	22.7 (19.5-24.3)	0.613
CD4^+^ T cell/μL, median (IQR)	19 (9-42)	17 (7-32)	26 (11-44)	0.502
HIV RNA, log_10_ copies/mL, median (IQR)	5.3 (4.9-5.8)	5 (4.5-5.6)	5.7 (5.1-6)	0.053

### The Plasma Metabolite Composition Is Different Between IRIS and Non-IRIS Groups

**Figure 1:**
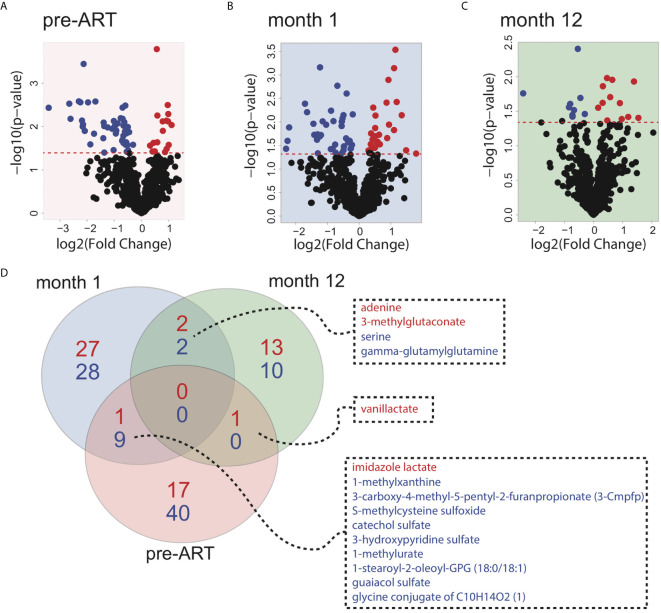
Global metabolite expression comparing IRIS with non-IRIS patients. Volcano plots depicting differences in metabolite levels between IRIS and non-IRIS patients are shown for the pre-ART **(A)**, month 1 **(B)**, and month 12 **(C)** post-ART time points. The significance threshold (*p*-value=0.05) is indicated by the red dashed line. Metabolites above the significance threshold are defined as the differentially expressed metabolites (DEMs). Each point represents an identified metabolite that is significantly upregulated (red) or downregulated (blue) in the IRIS group. **(D)** Venn diagram illustrating the numbers of differentially expressed metabolites is shown. Blue color represents the number of significantly downregulated metabolites comparing IRIS and non-IRIS groups, and red color represents the number of metabolites that are upregulated.

In order to understand the major metabolic alterations associated with IRIS development, we performed an untargeted metabolomics profiling that identified over 800 metabolites in all plasma samples from a library of over 5000 metabolites composed of amino acids, peptides, carbohydrates, lipids, energy molecules, nucleotides, cofactors and vitamins, and xenobiotics. First, we sought to investigate global differences in the metabolome of patients who developed IRIS compared to those who did not at each study time point. By using a threshold of p<0.05, 68 differentially expressed metabolites (DEMs) were identified at the pre-ART time point (**Figure 1A**). After 1 month of ART initiation or during the IRIS event, 69 DEMs were identified (**Figure 1B**). At the month 12 time point, the number of DEMs was reduced to 28 (**Figure 1C**). Detailed DEM identities and reported values are shown in **Supplementary Table 1**. Most of the DEMs were not shared between time points suggesting that the metabolome of IRIS patients is dynamic and changes over time (**Figure 1D**).

**Figure 2:**
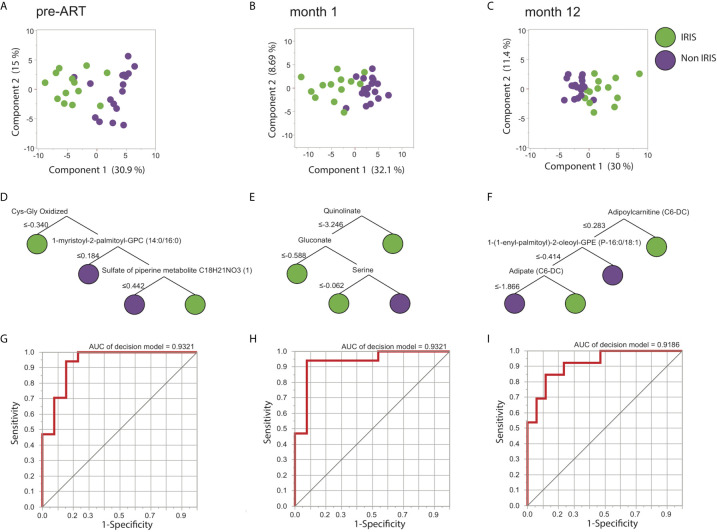
Differentially expressed metabolites (DEMs) prediction of IRIS. Identified DEMs are analyzed using PCA for the pre-ART **(A)**, month 1 **(B)**, and month 12 **(C)** time points. Decision tree models of the predicting metabolites are shown for the pre-ART **(D)**, month 1 **(E)**, and month 12 **(F)** time points. ROC analysis illustrating the decision tree discrimination power is shown in **(G)**, **(H)**, and **(I)** for the pre-ART, month 1, and month 12 time points respectively.

Following the identification of individual DEMs, we found that the DEMs were also able to distinguish most of IRIS and non-IRIS patients at each time point based on principal component analysis (PCA) (**Figures 2A–C**). We extended these findings by using an unsupervised hierarchical clustering, which further confirmed the discriminatory power of the DEMs for IRIS and non-IRIS patients at each time point (**Supplementary Figures 2A–C**). These findings demonstrated that IRIS patients already exhibited a distinct metabolic profile prior to the initiation of ART that persist through the IRIS event. Additionally, alterations in metabolic profile were still detectable up to a year after ART commencement although the differences were less conspicuous.

We next attempted to identify the most informative metabolites driving the distinct metabolic signatures for IRIS development at each time point. By using a machine learning approach named decision tree, we were able to select the minimum number of metabolites that differentiated IRIS from non-IRIS groups. Notably, at the pre-ART time point, three metabolites were defined as the most informative: oxidized cysteinyl-glycine (Cys-Gly Oxidized), 1-myristoyl-2-palmitoyl-GPC (14:0/16:0), and sulfate of piperine metabolite C_18_H_21_NO_3_ (**Figure 2D**). The combined result of these three metabolites could distinguish IRIS from non-IRIS group with high level of accuracy [area under the curve (AUC): 0.93, p<0.0001] (**Figures 2G**). At month 1, quinolinate, gluconate, and serine were the top analytes driving the distinction (AUC: 0.93, p<0.0001) (**Figures 2E, H**), and lastly at month 12, adipoylcarnitine (C6-DC), 1-(1-enyl-palmitoyl)-2-oleoyl-GPE (P-16:0/18:1), and adipate (C6-DC) accounted for the separation of the clinical groups (AUC: 0.92, p<0.0001) (**Figures 2F, I**).

### Altered Amino Acid and Lipid Metabolism Are Associated With IRIS Development

**Figure 3:**
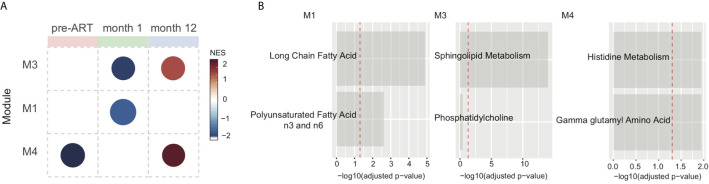
Metabolic pathway module analysis. Co-expressed pathway modules are obtained by using metabolite fold-difference values comparing IRIS with non-IRIS groups analyzed with the *CEMiTool* R package. **(A)** Module activity for each time point is shown. Color scale reflects the network enrichment score (NES) of each module. Red color indicates a high degree of representation and blue represents low degree of representation. **(B)** Metabolic pathways that define the three identified modules are depicted. Bar graphs show the –log_10_ adjusted *p*-value of fold difference from the metabolomics dataset. The vertical dashed red line indicates an adjusted *p*-value of 0.05.

We next sought to evaluate metabolic alterations on a pathway level that could differentiate IRIS from non-IRIS condition using a co-expressed pathway modules approach as described by Russo et al. (37). Cellular processes, especially cell metabolism, are highly complex and often regulated through many interacting networks. Therefore, the co-expression analysis would extract co-regulated metabolic pathways associated with IRIS pathogenesis that may have more biological relevance. Briefly, the co-expressed pathway analysis groups metabolites with similar fold-change measurements comparing IRIS to non-IRIS groups and their respective annotated metabolic pathways based on the Metabolon reference library into modules (M). Module activity or representation distinguishing IRIS and non-IRIS condition was indicated by the computed network enrichment scores (NES). From our analysis, we identified three annotated co-expressed modules with varying levels of representation at each study time points comparing IRIS with non-IRIS groups (**Figure 3A**). At the pre-ART time point, module 4 (M4) initially had low representation in patients who developed IRIS that became upregulated at month 12 (**Figure 3A**). M3 had low representation in the IRIS group at the month 1 time point followed by an increased representation at month 12 (**Figure 3A**). M1 was only identified to be underrepresented at the month 1 time point (**Figure 3A**). Furthermore, the identified modules encompassed metabolic pathways of amino acid and lipid metabolism (**Figure 3B**). Specifically, M1 was defined by metabolic pathways for long chain fatty acids and polyunsaturated fatty acids n3 and n6 metabolism. M3 included pathways for sphingolipid and phosphatidylcholine metabolism. The final module M4 was composed of histidine and gamma glutamyl amino acid metabolism (**Figure 3B**). By using the top metabolic pathway identified in each module in an unsupervised hierarchal clustering, we found that these three pathways were unable to distinctly cluster individual samples based on time points (**Supplementary Figure 2D**). These results demonstrated that although specific metabolic pathways were differentially and dynamically represented in IRIS at each study time point, the top module pathways alone were not sufficient to characterize the nuances in metabolic changes over time.

### Plasma Pro-Inflammatory Biomarkers Correlate With Metabolic Pathways in IRIS

**Figure 4:**
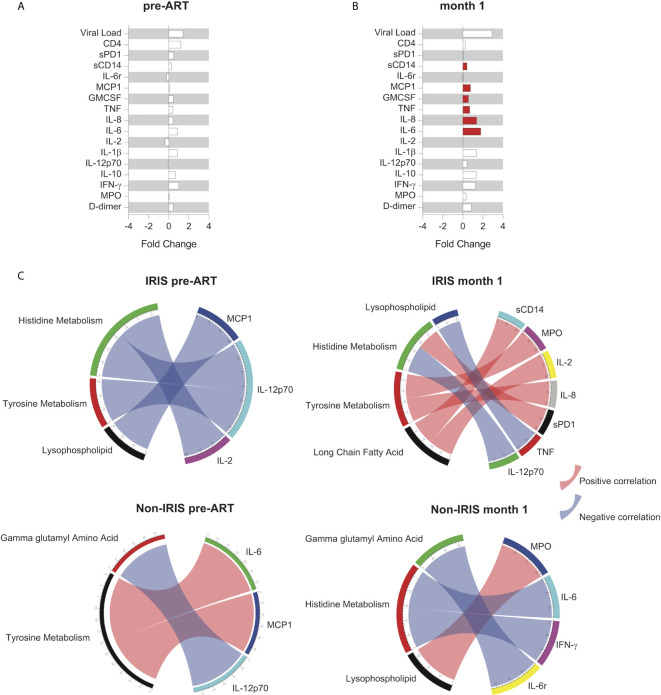
Association between metabolic pathways and systemic inflammation in IRIS. Fold change analysis between IRIS and non-IRIS groups of HIV viral load, total CD4^+^ T cell count, and levels of plasma biomarkers are shown for the pre-ART **(A)** and month 1 **(B)** time points. The statistically significant differences are shown in red bars. **(C)** Spearman correlations between levels of plasma biomarkers and normalized enrichment score from the co-expressed pathway modules at the pre-ART and month 1 time points for IRIS and non-IRIS groups are depicted. Blue arcs represent negative correlations, and red arcs represent positive correlations. Only statistically significant correlations with *p*-value <0.05 and correlation coefficient (r) above 0.7 or below -0.7 are portrayed.

Following the identification of DEMs and metabolic pathways unique to IRIS development, we investigated the association of metabolic pathways with plasma biomarker levels at the pre-ART and month 1 time points. At the pre-ART time point, there were no significant differences in the levels of measured plasma biomarkers between IRIS and non-IRIS patients (**Figure 4A**). After 1 month of ART initiation or during the IRIS event, biomarker measurements for soluble (s) CD14, MCP1, GMCSF, TNF, IL-8, and IL-6 were elevated in IRIS patients (**Figure 4B**).

Next, we correlated the metabolic pathway enrichment scores with plasma biomarker measurements using Spearman correlation matrices. Correlations achieving statistical significance (*p-*value <0.05, -0.7 < *r* > 0.7) were identified at the pre-ART and month 1 time points for both IRIS and non-IRIS groups (**Figure 4C**). We found that most of the significant correlations between plasma biomarkers and metabolic pathways occurred in the IRIS group during the IRIS event. The correlated metabolic pathways were in the lipid and amino acids metabolism families, indicating their potential regulatory involvement in IRIS pathogenesis and the implication of immune activation on lipid and amino acid metabolism disruptions. Specifically, lysophospholipid negatively correlated with TNF, and long chain fatty acids positively correlated with sCD14 and IL-2. Histidine metabolism positively correlated with sPD-1 and negatively correlated with IL-12p70. Lastly, tyrosine metabolism positively correlated with MPO and IL-8.

### Metabolome Complements the Plasma Biomarker Profile and the Transcriptome in a Multi-Omics Analysis to Characterize IRIS

Given the complexity of metabolic regulations in immune responses and IRIS pathogenesis, we performed an integrative multi-omics analysis of the metabolome, transcriptome, and plasma biomarkers comparing IRIS and non-IRIS patients. In total, there were 8 IRIS patients and 12 non-IRIS patients with full pairing of the three omics datasets (**Supplementary Table 1**). Through the MOFA pipeline, we identified four latent factors that had discriminatory power to differentiate IRIS and non-IRIS conditions based on the omics data input (**Supplementary Figure 3**). Latent factor 1 (LF1) exhibited the strongest differentiating power between IRIS and non-IRIS groups, whereas latent factor 4 had the lowest differentiating potential. Within LF1, plasma biomarkers and transcriptome showed higher degree of variance compare to the metabolome. We also determined the top 10 metabolites and plasma biomarkers, and the top 20 transcriptomic pathways that contributed to the differentiating potential of LF1 (**Supplementary Figures 3B–D**). Specifically, metabolites identified in LF1 were mostly amino acids or amino acids derivatives, and the transcriptomics pathways included RNA processing and modification, B cell signaling, and antimicrobial peptides transcription.

### Plasma Metabolomic Profiles Distinguish Mycobacterial IRIS From Other Types of IRIS

**Figure 5:**
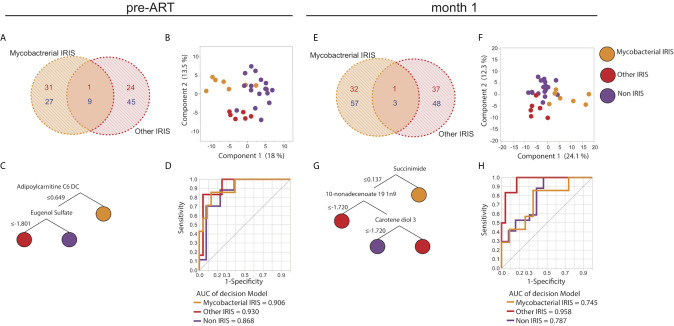
Unique metabolic signatures in different types of IRIS. Venn diagrams illustrating the number of unique and shared DEMs comparing mycobacterial IRIS to other types of IRIS at the pre-ART **(A)** and month 1 **(E)** time points are shown. DEMs were used in a PCA plot to depict dimensionally reduced data distribution of the mycobacterial IRIS, other types of IRIS, and non-IRIS groups at the pre-ART **(B)** and month 1 **(F)** time points. Decision tree models based on the DEMs are presented for the pre-ART **(C)** and month 1 **(G)** time points. ROC analysis of the metabolites identified by the decision trees were used to test discrimination power between each study group for pre-ART **(D)** and month 1 **(H)** time points. P-values for all the AUC measures were < 0.001.

As co-infections may have distinct impact on IRIS pathogenesis, we further compared the metabolic profiles of patients with different types of IRIS stratified based on mycobacterial IRIS and IRIS caused by other pathogens (**Supplementary Table 1**). Among the 17 HIV-infected non-IRIS individuals, six had mycobacterial co-infection (**Supplementary Table 1**). At baseline, the overall plasma metabolome based on all identified metabolites was not affected by the different co-infections (**Supplementary Figure 4**). Instead, the metabolic differences relied on specific metabolites and metabolic pathways. In particular, at the pre-ART time point, 58 metabolites were exclusively found in mycobacterial IRIS whereas 69 were uniquely modulated in the other types of IRIS group depicted in a Venn diagram (**Figure 5A**). Using the identified DEMs comparing mycobacterial IRIS, other types of IRIS, and non-IRIS groups, PCA and unsupervised hierarchical clustering analysis demonstrated that these three groups could be separated with minimal overlap (**Figure 5B** and **Supplementary Figures 5A–D, 6A, 6C**). The same Venn diagram and PCA analyses were repeated for the month 1 time point, and we found that DEMs were sufficient to distinguish all three groups (**Figures 5E, F** and **Supplementary Figures 5E–H, 6B, 6D**). We then employed the decision tree model and ROC analysis to select predictive metabolites at the pre-ART and month 1 time points. Results revealed that adipoylcarnitine C6 DC and eugenol sulfate could differentiate the outcome of mycobacterial IRIS, other types of IRIS, or non-IRIS conditions at the pre-ART time point (**Figures 5C, D**). At the month 1 time point, decision tree model determined succinimide, 10-nonadecenoate 19 1n9, and carotene-diol-3 exhibited discriminating power among the study groups (**Figure 5G**). ROC analysis identified an overall lower performance than that observed at the pre-ART time point, except for discriminating the other IRIS group from mycobacterial IRIS or non-IRIS groups (**Figure 5H**).

**Figure 6:**
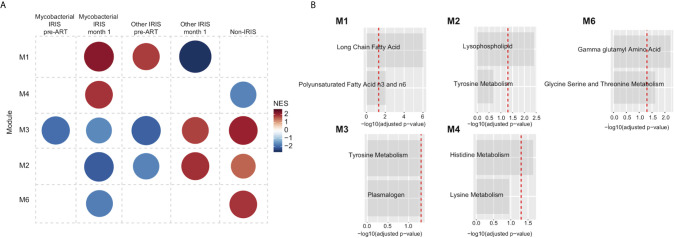
Metabolic pathway module analysis for different types of IRIS. **(A)** Co-expressed pathway modules are obtained by CEMiTool R package using the measured metabolite levels of mycobacterial IRIS, other types of IRIS, and non-IRIS groups at the pre-ART and month 1 time points. Module representation is reflected by the network enrichment scores (NES) **(B)** Annotated metabolic pathways that define each statistically significant module are shown.

Furthermore, co-expressed modules analysis was performed with same methodology used before for the comparison of mycobacterial IRIS, other types of IRIS and non-IRIS groups. There were 5 modules identified. Module representation for each group varied at different time points (**Figure 6A**). Metabolic pathways identified in each module belonged in the lipid and amino acid metabolism families, similar to those identified comparing IRIS and non-IRIS groups (**Figure 6B**). When the top pathway from each module was analyzed for each individual sample, we were unable to cluster the different group separately at the pre-ART and month 1 time points (**Supplementary Figures 6E, F**), which indicated these were not the only differentiating factors to discriminate study groups. Together, these findings revealed a perturbed host metabolism of IRIS that could potentially be associated with the underlying opportunistic pathogens and/or driven by differential gene regulation and inflammatory cytokine release.

## Discussion

It has been well characterized that IRIS is associated with aberrant innate and adaptive inflammatory responses to an underlying co-infection in PWH. Metabolic regulation of immune functions has gained growing attention in recent years. Here, we investigated the role of immunometabolism in IRIS pathogenesis through a comprehensive analysis of the plasma metabolome. We examined the longitudinal metabolic signatures across the pre-ART, IRIS event or 1 month post-ART equivalent, and 12 months post-ART time points. In addition, we compared various types of IRIS caused by different opportunistic infections. To our knowledge, this is the first study to present metabolomics analysis integrated with other biological parameters, such as plasma biomarkers and the transcriptome, to further decipher IRIS pathogenesis. Collectively, our findings identified potential lipid and amino acid metabolism perturbations in IRIS patients at the pre-ART and IRIS event time points, which could provide novel insight on predictors and therapeutic targets for IRIS.

We first identified more differentially expressed metabolites and correlations between metabolic pathways and inflammatory plasma biomarkers in IRIS patients prior to ART initiation and during the IRIS event. This finding not only reflected the association between cellular metabolism and the inflammatory manifestation during the IRIS event, but also revealed disrupted metabolism could be a predisposing factor for IRIS development. Following the resolution of IRIS inflammatory manifestations, metabolic differences diminished drastically by the month 12 time point between IRIS and non-IRIS groups. Although a small number of DEMs, mostly belonging to amino acid and nucleotide metabolism pathways, were still detectable at the month 12 time point, these differences could be contributed to residual inflammation, lifestyle, or medication. Furthermore, the detection of metabolic changes at the pre-ART and IRIS event in the current study was consistent with our previous findings of increased metabolic activity accompanied with higher glucose transporter expression on CD4^+^ T cells and monocytes in IRIS patients prior to ART initiation and during the IRIS event (20). IRIS pathogenesis has been characterized by exaggerated immune activation including elevated plasma pro-inflammatory biomarkers (6, 9, 16), skewed inflammatory monocyte population (6), and robust polyfunctional antigen-specific CD4^+^ T cell responses (3, 9, 12). In the context of metabolism and immune activation, infection by invading pathogen and T cell activation through T cell receptor ligation trigger metabolic reprogramming defined by increased glycolysis and modified mitochondria function in T-helper 1 cells, monocytes, and macrophages (22, 27, 28, 43). Metabolic reprogramming, in turn, is also essential to effector function in immune cells as blocking glycolysis and mitochondria function can inhibit immune responses (22, 27). Metabolites can also act as immunological mediators to regulate TCR activation, epigenetic modifications, and expression of transcription factors or cytokines (23, 44, 45). Consequently, disturbance of the metabolic machinery and the generation of metabolites can have a profound effect on immune activation and immune activation may also induce metabolic changes. Therefore, our observation of an altered plasma metabolite profile prior to ART initiation and during the IRIS event conform with the established connection between metabolic regulations and immune function.

The use of metabolite decision tree models, co-expressed pathway module analysis, and plasma biomarker correlations revealed amino acid and lipid metabolites as the predominant variables that differentiated IRIS from non-IRIS conditions. First, amino acids are known as the building blocks for protein synthesis and protein-mediated inflammatory signaling (46, 47). In the pre-ART decision tree, oxidized cysteinyl-glycine (cys-gly), a dipeptide composed of amino acid glycine with an attached L-cysteinyl group, was the top metabolite to distinguish IRIS from non-IRIS conditions. Cys-gly is an intermediate metabolite in the glutathione metabolism pathway that can regulate oxidative stress (48). Elevated level of cys-gly has been shown to be associated with chronic immune activation in HIV-infected individuals on ART (33). Therefore, the level of cys-gly at the pre-ART time point could be an indication for heightened immune activation and predisposes patients for the development of IRIS. In the month 1 time point decision tree, another amino acid derivative quinolinate was the first metabolite to differentiate IRIS from non-IRIS condition. The catabolism of tryptophan to kynurenine and subsequent downstream metabolites including quinolinate have been implicated to have immunoregulatory roles. Specifically, quinolinate is involved in neurodegenerative diseases associated with AIDS (49, 50). Increased activity of tryptophan metabolism, quantified by elevated plasma ratio of kynurenine to tryptophan (KT ratio), has also been observed in HIV patients and is associated HIV disease severity and higher mortality rate (51, 52). In addition, proinflammatory cytokine IFN-γ induces the activity of the rate-limiting enzyme indoleamine-2,3-deoxygenase (IDO) in tryptophan metabolism (53). Thus, the tryptophan/kynurenine pathway and associated metabolites could play a role in IRIS pathogenesis as IRIS is characterized by exaggerated immune responses and robust IFN-γ production by antigen-specific T cells.

In addition to the decision tree predictive metabolites, correlations between metabolic pathways and plasma biomarkers have revealed that amino acids histidine and tyrosine metabolism may influence the inflammatory status of IRIS. In particular, histidine metabolism was negatively correlated with pro-inflammatory cytokine IL-12p70 and positively correlated with inhibitory marker soluble PD-1. Reduced histidine metabolism has been described in chronic inflammatory disease such as systemic lupus erythematosus (30, 31, 54). IFN-γ has also been demonstrated to upregulate the histidine catabolizing enzymes resulting in the depletion of free histidine (55). Therefore, the correlations between histidine metabolism and two plasma biomarkers essential for T cell activation emphasized the potential inhibitory effect of histidine metabolism in immune activation and IRIS pathogenesis. Tyrosine metabolism was found to positively correlate with both myeloperoxidase (MPO) and IL-8, which are essential for neutrophil responses during an infection. The induction of IL-8 facilitates innate immune responses to an infection and mediate neutrophil chemotaxis (56). Neutrophils at the inflammatory sites secrete enzyme MPO to generate antimicrobial agent hypochlorous acid and the enzymatic activity of MPO can be enhanced by free tyrosine in the extracellular space (57). As a result, the correlation of tyrosine metabolism with both IL-8 and MPO provide insight on the role of neutrophils in mediating the aberrant inflammatory responses in IRIS.

Conversely, lipid metabolism is also crucial for immune cell activation to provide both cell membrane structure and high energy fuel to maintain memory responses (58, 59). We have identified polyunsaturated fatty acids (PUFAs), long-chain fatty acids (LCFAs), sphingolipids, phosphatidylcholines (PCs), and lysophospholipids (LPLs) metabolism pathways to be differentially expressed in IRIS. Another recent TB-IRIS metabolomics study also described an altered plasma lipid metabolism signature in TB-IRIS patients and highlighted the distinct differences of PUFAs (34). PUFAs and PUFA-derived lipid mediators including prostaglandins and lipoxins exhibit immunoregulatory functions in both the innate and adaptive immune systems to modulate T cell activation, cytokine production, cell membrane permeability, and intracellular signaling (34, 60). In addition, dietary supplementation of PUFAs were found to be effective in modulating inflammatory responses in inflammatory bowel disease (IBD) animal models (61). LCFAs are also crucial to support T cell effector differentiation and function. When LCFA oxidation is irreversibly inhibited, T cell differentiation and memory T cell secondary activation were drastically hindered (62, 63). Lastly, sphingolipids, PCs, and LPLs are membrane lipids that not only provide membrane structure, but also function as signaling molecules to elicit host immune responses in autoimmune and cardiovascular diseases (64, 65). Sphingolipids have been targeted as therapeutic measures in both asthma and IBD to reduce the levels of pro-inflammatory cytokines and alleviate inflammation (66). Together, our results and previously published studies provide evidence for the intimate involvement of fatty acid metabolism in inflammation and IRIS pathogenesis.

In an attempt to further delineate the implication of metabolomics in IRIS pathogenesis, we employed the MOFA model to incorporate the metabolome, transcriptome, and plasma biomarker profile. We have demonstrated the success of this approach previously in settings such as TB, diabetes, and leishmaniasis, providing important insights into the pathogenesis of these pathological conditions (40, 41, 67). The IRIS metabolome provided complementary information that expanded our understanding of the profound immune activation observed in IRIS patients. Within MOFA, the metabolic and transcriptomic pathways variance was largely driven by amino acid metabolism and protein translation machineries, which mirrored the other findings in this study highlighting the crucial role of amino acids in immune activation. Cellular metabolism is often regulated by redundant pathways to maintain homeostasis when encountering disruptive signals. This could explain the less robust variance observed in IRIS plasma metabolome compared to the transcriptome and the plasma biomarker profile, where protein translation and concentration could be more direct cellular readouts.

Finally, several additional key features that can influence the metabolome include microbial invasion and sex (68, 69). We postulate that the unique metabolic profile of different types of IRIS are largely driven by the underlying co-infection as has been previously demonstrated in TB (32, 70, 71). In particular, human monocyte-derived macrophages infected with *M. tuberculosis* could induce a shift from oxidative phosphorylation to aerobic glycolysis (70). Another plasma metabolomics study showed significantly different levels of lipid metabolites detected in patients with active TB disease (32). Lastly, a multi-omics study integrating plasma metabolome and cell transcriptome identified signatures associated with TB progression in glutathione pathway, sphingolipid pathway, and tRNA processing (39). Thus, our findings contrasting different types of IRIS likely reflect the distinct metabolic signature influenced by the co-infection pathogen. The influence of sex on the metabolome has been explored in several previous studies. By using an untargeted metabolomics approach, differences in plasma or serum metabolite composition including lipid steroids and derivative metabolites, branched-chain amino acids used for muscle building, and short-chain fatty acids could be detected contrasting age-matched men and women groups (72–74). Findings from these studies highlight the importance of sex-matched study groups to ensure that metabolomics results are not influenced by confounding factors. In the current study, we have proportionate numbers of female study participants in the IRIS and non-IRIS groups. In addition, based on PCA analysis of all identified metabolites, although limited by a small sample size, the effect of sex on the metabolome was not different comparing IRIS and non-IRIS groups at the three study time points (**Supplementary Figure 7**).

There were several limitations in our study. First, we had a relatively small sample size especially of non-mycobacterial IRIS. In addition, we were restricted by the number of fully matched samples to perform the multi-omics analysis at each time point. Second, the plasma metabolome embodies extracellular metabolites produced from all cell types throughout the body. As a result, we cannot determine the source of metabolite production or consumption. Third, we lack extensive *in vitro* validation for the computationally identified metabolic signatures, which could serve as predictive or therapeutic targets. Lastly, we lack the inclusion of HIV uninfected healthy donors as another comparator group. Although our IRIS and non-IRIS group comparisons have been performed within a homogenous HIV infection background, an uninfected control group with similar demographics could have provide the overall framework of the healthy plasma metabolome and a better depiction of the metabolic contributions to the pathological state of IRIS.

In conclusion, IRIS was associated with a distinct plasma metabolomics profile characterized by perturbed lipid and amino acid metabolism at the pre-ART and IRIS event time points. This study expanded our understanding for the role of cellular metabolism in IRIS pathogenesis and complemented our previously findings of glycolytic shift by FDG-PET scan and *in vitro* measurements of glucose transporter expression on monocytes and T lymphocytes (20). Thus, metabolic reprogramming could fuel the dysregulated immune activation in IRIS and metabolic pathways may serve as novel targets for preventative and therapeutic measures in inflammatory complications.

## Data Availability Statement

The RNA sequencing dataset presented in this study can be found in online repository at the GEO database GSE173697: https://www.ncbi.nlm.nih.gov/geo/query/acc.cgi?acc=GSE173697. The normalized metabolomics raw values can be found in the **Supplementary Material**.

## Ethics Statement

The studies involving human participants were reviewed and approved by NIH Institutional Review Board. The patients/participants provided their written informed consent to participate in this study.

## Author Contributions

LP, KF, BA, and IS designed the study, contributed to data collection and analysis, and drafted the final manuscript. KF, RT, and BA processed data and generated data visualization. AR performed plasma biomarker measurements. ED performed whole blood RNA sequencing. FG, EL, MM, AL, and IS coordinated and provided clinical care. All authors contributed to the article and approved the submitted version.

## Funding

This work is supported by the Intramural Research Program of the National Institute of Allergy and Infectious Diseases at the National Institutes of Health (NIH).

## Conflict of Interest

AR was employed by Leidos Biomedical Research, Inc.

The authors remaining declare that the research was conducted in the absence of any commercial or financial relationships that could be construed as a potential conflict of interest.

## References

[1] French MA. HIV/AIDS: Immune Reconstitution Inflammatory Syndrome: A Reappraisal. Clin Infect Dis (2009) 48(1):101–7. 10.1086/595006 19025493

[2] Barber DL Andrade BB Sereti I Sher A. Immune Reconstitution Inflammatory Syndrome: The Trouble With Immunity When You had None. Nat Rev Microbiol (2012) 10(2):150–6. 10.1038/nrmicro2712 PMC3507517 22230950

[3] Hsu DC Breglio KF Pei L Wong CS Andrade BB Sheikh V. Emergence of Polyfunctional Cytotoxic Cd4+ T Cells in Mycobacterium Avium Immune Reconstitution Inflammatory Syndrome in Human Immunodeficiency Virus-Infected Patients. Clin Infect Dis (2018) 67(3):437–46. 10.1093/cid/ciy016 PMC6248720 29538651

[4] Sereti I Sheikh V Shaffer D Phanuphak N Gabriel E Wang J. Prospective International Study of Incidence and Predictors of Immune Reconstitution Inflammatory Syndrome and Death in People Living With Human Immunodeficiency Virus and Severe Lymphopenia. Clin Infect Dis (2020) 71(3):652–60. 10.1093/cid/ciz877 PMC7384325 31504347

[5] Vinhaes CL Araujo-Pereira M Tiburcio R Cubillos-Angulo JM Demitto FO Akrami KM. Systemic Inflammation Associated With Immune Reconstitution Inflammatory Syndrome in Persons Living With HIV. Life (Basel) (2021) 11(1):65. 10.3390/life11010065 33477581 PMC7831327

[6] Andrade BB Singh A Narendran G Schechter ME Nayak K Subramanian S. Mycobacterial Antigen Driven Activation of CD14++CD16– Monocytes Is a Predictor of Tuberculosis-Associated Immune Reconstitution Inflammatory Syndrome. PloS Pathog (2014) 10(10):e1004433. 10.1371/journal.ppat.1004433 25275318 PMC4183698

[7] Namale PE Abdullahi LH Fine S Kamkuemah M Wilkinson RJ Meintjes G. Paradoxical TB-IRIS in HIV-Infected Adults: A Systematic Review and Meta-Analysis. Future Microbiol (2015) 10(6):1077–99. 10.2217/fmb.15.9 26059627

[8] Meintjes G Wilkinson RJ Morroni C Pepper DJ Rebe K Rangaka MX. Randomized Placebo-Controlled Trial of Prednisone for Paradoxical Tuberculosis-Associated Immune Reconstitution Inflammatory Syndrome. AIDS (2010) 24(15):2381–90. 10.1097/QAD.0b013e32833dfc68 PMC2940061 20808204

[9] Antonelli LR Mahnke Y Hodge JN Porter BO Barber DL DerSimonian R. Elevated Frequencies of Highly Activated CD4+ T Cells in HIV+ Patients Developing Immune Reconstitution Inflammatory Syndrome. Blood (2010) 116(19):3818–27. 10.1182/blood-2010-05-285080 PMC2981537 20660788

[10] Barber DL Andrade BB McBerry C Sereti I Sher A. Role of IL-6 in Mycobacterium Avium–Associated Immune Reconstitution Inflammatory Syndrome. J Immunol (2014) 192(2):676–82. 10.4049/jimmunol.1301004 PMC3947368 24337386

[11] Barber DL Mayer-Barber KD Antonelli LRV Wilson MS White S Caspar P. Th1-driven Immune Reconstitution Disease in Mycobacterium Avium–Infected Mice. Blood (2010) 116(18):3485–93. 10.1182/blood-2010-05-286336 PMC2981475 20656932

[12] Mahnke YD Greenwald JH DerSimonian R Roby G Antonelli LR Sher A. Selective Expansion of Polyfunctional Pathogen-Specific CD4(+) T Cells in HIV-1-infected Patients With Immune Reconstitution Inflammatory Syndrome. Blood (2012) 119(13):3105–12. 10.1182/blood-2011-09-380840 PMC3321870 22219223

[13] Ravimohan S Tamuhla N Nfanyana K Steenhoff AP Letlhogile R Frank I. Robust Reconstitution of Tuberculosis-Specific Polyfunctional CD4+ T-Cell Responses and Rising Systemic Interleukin 6 in Paradoxical Tuberculosis-Associated Immune Reconstitution Inflammatory Syndrome. Clin Infect Dis (2016) 62(6):795–803. 10.1093/cid/civ978 26611774 PMC4772844

[14] Meintjes G Skolimowska KH Wilkinson KA Matthews K Tadokera R Conesa-Botella A. Corticosteroid-Modulated Immune Activation in the Tuberculosis Immune Reconstitution Inflammatory Syndrome. Am J Respir Crit Care Med (2012) 186(4):369–77. 10.1164/rccm.201201-0094OC PMC3443811 22700860

[15] Tran HT Van den Bergh R Vu TN Laukens K Worodria W Loembe MM. The Role of Monocytes in the Development of Tuberculosis-Associated Immune Reconstitution Inflammatory Syndrome. Immunobiology (2014) 219(1):37–44. 10.1016/j.imbio.2013.07.004 23958034

[16] Grant PM Komarow L Lederman MM Pahwa S Zolopa AR Andersen J. Elevated Interleukin 8 and T-helper 1 and T-helper 17 Cytokine Levels Prior to Antiretroviral Therapy in Participants Who Developed Immune Reconstitution Inflammatory Syndrome During ACTG A5164. J Infect Dis (2012) 206(11):1715–23. 10.1093/infdis/jis604 PMC3488199 23002445

[17] Boulware DR Hullsiek KH Puronen CE Rupert A Baker JV French MA. Higher Levels of CRP, D-Dimer, IL-6, and Hyaluronic Acid Before Initiation of Antiretroviral Therapy (ART) are Associated With Increased Risk of AIDS or Death. J Infect Dis (2011) 203(11):1637–46. 10.1093/infdis/jir134 PMC3096784 21592994

[18] Boulware DR Meya DB Bergemann TL Wiesner DL Rhein J Musubire A. Clinical Features and Serum Biomarkers in HIV Immune Reconstitution Inflammatory Syndrome After Cryptococcal Meningitis: A Prospective Cohort Study. PloS Med (2010) 7(12):e1000384. 10.1371/journal.pmed.1000384 21253011 PMC3014618

[19] Hsu DC Faldetta KF Pei L Sheikh V Utay NS Roby G. A Paradoxical Treatment for a Paradoxical Condition: Infliximab Use in Three Cases of Mycobacterial Iris. Clin Infect Dis (2016) 62(2):258–61. 10.1093/cid/civ841 PMC4690485 26394669

[20] Hammoud DA Boulougoura A Papadakis GZ Wang J Dodd LE Rupert A. Increased Metabolic Activity on 18F-Fluorodeoxyglucose Positron Emission Tomography–Computed Tomography in Human Immunodeficiency Virus–Associated Immune Reconstitution Inflammatory Syndrome. Clin Infect Dis (2019) 68(2):229–38. 10.1093/cid/ciy454 PMC6321853 30215671

[21] Akkaya B Roesler AS Miozzo P Theall BP Al Souz J Smelkinson MG. Increased Mitochondrial Biogenesis and Reactive Oxygen Species Production Accompany Prolonged Cd4(+) T Cell Activation. J Immunol (2018) 201(11):3294–306. 10.4049/jimmunol.1800753 PMC6246812 30373851

[22] Buck MD O’Sullivan D Geltink RIK Curtis JD Chang C-H Sanin DE. Mitochondrial Dynamics Controls T Cell Fate Through Metabolic Programming. Cell (2016) 166(1):63–76. 10.1016/j.cell.2016.05.035 27293185 PMC4974356

[23] Macintyre AN Gerriets VA Nichols AG Michalek RD Rudolph MC Deoliveira D. The Glucose Transporter Glut1 is Selectively Essential for CD4 T Cell Activation and Effector Function. Cell Metab (2014) 20(1):61–72. 10.1016/j.cmet.2014.05.004 24930970 PMC4079750

[24] Peng M Yin N Chhangawala S Xu K Leslie CS Li MO. Aerobic Glycolysis Promotes T Helper 1 Cell Differentiation Through an Epigenetic Mechanism. Science (2016) 354(6311):481–4. 10.1126/science.aaf6284 PMC5539971 27708054

[25] Ricciardi S Manfrini N Alfieri R Calamita P Crosti MC Gallo S. The Translational Machinery of Human Cd4+ T Cells Is Poised for Activation and Controls the Switch From Quiescence to Metabolic Remodeling. Cell Metab (2018) 28(6):895–906.e5. 10.1016/j.cmet.2018.08.009 30197303 PMC6773601

[26] Sena LA Li S Jairaman A Prakriya M Ezponda T Hildeman DA. Mitochondria are Required for Antigen-Specific T Cell Activation Through Reactive Oxygen Species Signaling. Immunity (2013) 38(2):225–36. 10.1016/j.immuni.2012.10.020 PMC3582741 23415911

[27] Chang CH Curtis JD Maggi LB Jr. Faubert B Villarino AV O’Sullivan D. Posttranscriptional Control of T Cell Effector Function by Aerobic Glycolysis. Cell (2013) 153(6):1239–51. 10.1016/j.cell.2013.05.016 PMC3804311 23746840

[28] O’Neill LA Pearce EJ. Immunometabolism Governs Dendritic Cell and Macrophage Function. J Exp Med (2016) 213(1):15–23. 10.1084/jem.20151570 26694970 PMC4710204

[29] Al-Mubarak R Vander Heiden J Broeckling CD Balagon M Brennan PJ Vissa VD. Serum Metabolomics Reveals Higher Levels of Polyunsaturated Fatty Acids in Lepromatous Leprosy: Potential Markers for Susceptibility and Pathogenesis. PloS Negl Trop Dis (2011) 5(9):e1303. 10.1371/journal.pntd.0001303 21909445 PMC3167790

[30] Bengtsson AA Trygg J Wuttge DM Sturfelt G Theander E Donten M. Metabolic Profiling of Systemic Lupus Erythematosus and Comparison With Primary Sjogren’s Syndrome and Systemic Sclerosis. PloS One (2016) 11(7):e0159384. 10.1371/journal.pone.0159384 27441838 PMC4956266

[31] Perl A Hanczko R Lai Z-W Oaks Z Kelly R Borsuk R. Comprehensive Metabolome Analyses Reveal N-Acetylcysteine-Responsive Accumulation of Kynurenine in Systemic Lupus Erythematosus: Implications for Activation of the Mechanistic Target of Rapamycin. Metabolomics (2015) 11(5):1157–74. 10.1007/s11306-015-0772-0 PMC4559110 26366134

[32] Collins JM Walker DI Jones DP Tukvadze N Liu KH Tran VT. High-Resolution Plasma Metabolomics Analysis to Detect Mycobacterium Tuberculosis-Associated Metabolites That Distinguish Active Pulmonary Tuberculosis in Humans. PloS One (2018) 13(10):e0205398. 10.1371/journal.pone.0205398 30308073 PMC6181350

[33] Chettimada S Lorenz DR Misra V Dillon ST Reeves RK Manickam C. Exosome Markers Associated With Immune Activation and Oxidative Stress in HIV Patients on Antiretroviral Therapy. Sci Rep (2018) 8(1):7227. 10.1038/s41598-018-25515-4 29740045 PMC5940833

[34] Silva CAM Graham B Webb K Ashton LV Harton M Luetkemeyer AF. A Pilot Metabolomics Study of Tuberculosis Immune Reconstitution Inflammatory Syndrome. Int J Infect Dis (2019) 84:30–8. 10.1016/j.ijid.2019.04.015 PMC6613934 31009738

[35] Evans AM DeHaven CD Barrett T Mitchell M Milgram E. Integrated, Nontargeted Ultrahigh Performance Liquid Chromatography/Electrospray Ionization Tandem Mass Spectrometry Platform for the Identification and Relative Quantification of the Small-Molecule Complement of Biological Systems. Anal Chem (2009) 81(16):6656–67. 10.1021/ac901536h 19624122

[36] Espindola MS Lima LJ Soares LS Cacemiro MC Zambuzi FA de Souza Gomes M. Dysregulated Immune Activation in Second-Line Haart HIV+ Patients Is Similar to That of Untreated Patients. PloS One (2015) 10(12):e0145261. 10.1371/journal.pone.0145261 26684789 PMC4684276

[37] Russo PST Ferreira GR Cardozo LE Bürger MC Arias-Carrasco R Maruyama SR. CemiTool: A Bioconductor Package for Performing Comprehensive Modular Co-Expression Analyses. BMC Bioinf (2018) 19(1):56. 10.1186/s12859-018-2053-1 PMC5819234 29458351

[38] Vinhaes CL Oliveira-de-Souza D Silveira-Mattos PS Nogueira B Shi R Wei W. Changes in Inflammatory Protein and Lipid Mediator Profiles Persist After Antitubercular Treatment of Pulmonary and Extrapulmonary Tuberculosis: A Prospective Cohort Study. Cytokine (2019) 123:154759. 10.1016/j.cyto.2019.154759 31226436 PMC6739167

[39] Duffy FJ Weiner J 3rd Hansen S Tabb DL Suliman S Thompson E. Immunometabolic Signatures Predict Risk of Progression to Active Tuberculosis and Disease Outcome. Front Immunol (2019) 10:527. 10.3389/fimmu.2019.00527 30967866 PMC6440524

[40] Cubillos-Angulo JM Vinhaes CL Fukutani ER Albuquerque VVS Queiroz ATL Andrade BB. In Silico Transcriptional Analysis of mRNA and miRNA Reveals Unique Biosignatures That Characterizes Different Types of Diabetes. PloS One (2020) 15(9):e0239061. 10.1371/journal.pone.0239061 32956382 PMC7505453

[41] Malta-Santos H Fukutani KF Sorgi CA Queiroz ATL Nardini V Silva J. Multi-Omic Analyses of Plasma Cytokines, Lipidomics, and Transcriptomics Distinguish Treatment Outcomes in Cutaneous Leishmaniasis. iScience (2020) 23(12):101840. 10.1016/j.isci.2020.101840 33313489 PMC7721649

[42] Argelaguet R Velten B Arnol D Dietrich S Zenz T Marioni JC. Multi-Omics Factor Analysis-A Framework for Unsupervised Integration of Multi-Omics Data Sets. Mol Syst Biol (2018) 14(6):e8124. 10.15252/msb.20178124 29925568 PMC6010767

[43] Cheng SC Quintin J Cramer RA Shepardson KM Saeed S Kumar V. mTOR- and HIF-1Alpha-Mediated Aerobic Glycolysis as Metabolic Basis for Trained Immunity. Science (2014) 345(6204):1250684. 10.1126/science.1250684 25258083 PMC4226238

[44] Wang R Dillon CP Shi LZ Milasta S Carter R Finkelstein D. The Transcription Factor Myc Controls Metabolic Reprogramming Upon T Lymphocyte Activation. Immunity (2011) 35(6):871–82. 10.1016/j.immuni.2011.09.021 PMC3248798 22195744

[45] Delgoffe GM Powell JD. Sugar, Fat, and Protein: New Insights Into What T Cells Crave. Curr Opin Immunol (2015) 33:49–54. 10.1016/j.coi.2015.01.015 25665466 PMC4397153

[46] Tannahill GM Curtis AM Adamik J Palsson-McDermott EM McGettrick AF Goel G. Succinate is an Inflammatory Signal That Induces IL-1beta Through HIF-1alpha. Nature (2013) 496(7444):238–42. 10.1038/nature11986 PMC4031686 23535595

[47] Hosios AM Hecht VC Danai LV Johnson MO Rathmell JC Steinhauser ML. Amino Acids Rather Than Glucose Account for the Majority of Cell Mass in Proliferating Mammalian Cells. Dev Cell (2016) 36(5):540–9. 10.1016/j.devcel.2016.02.012 PMC4766004 26954548

[48] Muller T Muhlack S. Cysteinyl-Glycine Reduction as Marker for Levodopa-Induced Oxidative Stress in Parkinson’s Disease Patients. Mov Disord (2011) 26(3):543–6. 10.1002/mds.23384 21462263

[49] Valle M Price RW Nilsson A Heyes M Verotta D. CSF Quinolinic Acid Levels are Determined by Local HIV Infection: Cross-Sectional Analysis and Modelling of Dynamics Following Antiretroviral Therapy. Brain (2004) 127(Pt 5):1047–60. 10.1093/brain/awh130 15013955

[50] Moffett JR Arun P Puthillathu N Vengilote R Ives JA Badawy AA. Quinolinate as a Marker for Kynurenine Metabolite Formation and the Unresolved Question of NAD(+) Synthesis During Inflammation and Infection. Front Immunol (2020) 11:31. 10.3389/fimmu.2020.00031 32153556 PMC7047773

[51] Byakwaga H Boum Y Huang Y Muzoora C Kembabazi A Weiser SD. The Kynurenine Pathway of Tryptophan Catabolism, CD4+ T-Cell Recovery, and Mortality Among HIV-Infected Ugandans Initiating Antiretroviral Therapy. J Infect Dis (2014) 210(3):383–91. 10.1093/infdis/jiu115 PMC4148610 24585899

[52] Lee S Byakwaga H Boum Y Burdo TH Williams KC Lederman MM. Immunologic Pathways That Predict Mortality in HIV-Infected Ugandans Initiating Antiretroviral Therapy. J Infect Dis (2017) 215(8):1270–4. 10.1093/infdis/jix113 PMC5853335 28329310

[53] Yap SH Abdullah NK McStea M Takayama K Chong ML Crisci E. HIV/Human Herpesvirus Co-Infections: Impact on Tryptophan-Kynurenine Pathway and Immune Reconstitution. PloS One (2017) 12(10):e0186000. 10.1371/journal.pone.0186000 29016635 PMC5633182

[54] Perl A. Review: Metabolic Control of Immune System Activation in Rheumatic Diseases. Arthritis Rheumatol (2017) 69(12):2259–70. 10.1002/art.40223 PMC5711528 28841779

[55] Dwivedy A Ashraf A Jha B Kumar D Agarwal N Biswal BK. De Novo Histidine Biosynthesis Protects Mycobacterium Tuberculosis From Host IFN-Gamma Mediated Histidine Starvation. Commun Biol (2021) 4(1):410. 10.1038/s42003-021-01926-4 33767335 PMC7994828

[56] Skov L Beurskens FJ Zachariae CO Reitamo S Teeling J Satijn D. IL-8 as Antibody Therapeutic Target in Inflammatory Diseases: Reduction of Clinical Activity in Palmoplantar Pustulosis. J Immunol (2008) 181(1):669–79. 10.4049/jimmunol.181.1.669 18566434

[57] Vlasova II Sokolov AV Arnhold J. The Free Amino Acid Tyrosine Enhances the Chlorinating Activity of Human Myeloperoxidase. J Inorg Biochem (2012) 106(1):76–83. 10.1016/j.jinorgbio.2011.09.018 22112843

[58] Pearce EL Walsh MC Cejas PJ Harms GM Shen H Wang LS. Enhancing CD8 T-Cell Memory by Modulating Fatty Acid Metabolism. Nature (2009) 460(7251):103–7. 10.1038/nature08097 PMC2803086 19494812

[59] Lee J Walsh MC Hoehn KL James DE Wherry EJ Choi Y. Regulator of Fatty Acid Metabolism, Acetyl Coenzyme a Carboxylase 1, Controls T Cell Immunity. J Immunol (2014) 192(7):3190–9. 10.4049/jimmunol.1302985 PMC3965631 24567531

[60] McMurray DN Jolly CA Chapkin RS. Effects of Dietary N-3 Fatty Acids on T Cell Activation and T Cell Receptor-Mediated Signaling in a Murine Model. J Infect Dis (2000) 182(Suppl 1):S103–7. 10.1086/315909 10944491

[61] Michalak A Mosinska P Fichna J. Polyunsaturated Fatty Acids and Their Derivatives: Therapeutic Value for Inflammatory, Functional Gastrointestinal Disorders, and Colorectal Cancer. Front Pharmacol (2016) 7:459. 10.3389/fphar.2016.00459 27990120 PMC5131004

[62] Raud B Roy DG Divakaruni AS Tarasenko TN Franke R Ma EH. Etomoxir Actions on Regulatory and Memory T Cells Are Independent of Cpt1a-Mediated Fatty Acid Oxidation. Cell Metab (2018) 28(3):504–15.e7. 10.1016/j.cmet.2018.06.002 30043753 PMC6747686

[63] van der Windt GJ O’Sullivan D Everts B Huang SC Buck MD Curtis JD. CD8 Memory T Cells Have a Bioenergetic Advantage That Underlies Their Rapid Recall Ability. Proc Natl Acad Sci USA (2013) 110(35):14336–41. 10.1073/pnas.1221740110 PMC3761631 23940348

[64] Edsfeldt A Duner P Stahlman M Mollet IG Asciutto G Grufman H. Sphingolipids Contribute to Human Atherosclerotic Plaque Inflammation. Arterioscler Thromb Vasc Biol (2016) 36(6):1132–40. 10.1161/ATVBAHA.116.305675 27055903

[65] Maceyka M Spiegel S. Sphingolipid Metabolites in Inflammatory Disease. Nature (2014) 510(7503):58–67. 10.1038/nature13475 24899305 PMC4320971

[66] Nixon GF. Sphingolipids in Inflammation: Pathological Implications and Potential Therapeutic Targets. Br J Pharmacol (2009) 158(4):982–93. 10.1111/j.1476-5381.2009.00281.x PMC2785521 19563535

[67] Dutta NK Tornheim JA Fukutani KF Paradkar M Tiburcio RT Kinikar A. Integration of Metabolomics and Transcriptomics Reveals Novel Biomarkers in the Blood for Tuberculosis Diagnosis in Children. Sci Rep (2020) 10(1):19527. 10.1038/s41598-020-75513-8 33177551 PMC7658223

[68] Keshavarz M Solaymani-Mohammadi F Namdari H Arjeini Y Mousavi MJ Rezaei F. Metabolic Host Response and Therapeutic Approaches to Influenza Infection. Cell Mol Biol Lett (2020) 25:15. 10.1186/s11658-020-00211-2 32161622 PMC7059726

[69] Lovewell RR Sassetti CM VanderVen BC. Chewing the Fat: Lipid Metabolism and Homeostasis During M. Tuberculosis Infection. Curr Opin Microbiol (2016) 29:30–6. 10.1016/j.mib.2015.10.002 26544033

[70] Gleeson LE Sheedy FJ Palsson-McDermott EM Triglia D O’Leary SM O’Sullivan MP. Cutting Edge: Mycobacterium Tuberculosis Induces Aerobic Glycolysis in Human Alveolar Macrophages That Is Required for Control of Intracellular Bacillary Replication. J Immunol (2016) 196(6):2444–9. 10.4049/jimmunol.1501612 26873991

[71] Vrieling F Kostidis S Spaink HP Haks MC Mayboroda OA Ottenhoff THM. Analyzing the Impact of Mycobacterium Tuberculosis Infection on Primary Human Macrophages by Combined Exploratory and Targeted Metabolomics. Sci Rep (2020) 10(1):7085. 10.1038/s41598-020-62911-1 32341411 PMC7184630

[72] Rist MJ Roth A Frommherz L Weinert CH Kruger R Merz B. Metabolite Patterns Predicting Sex and Age in Participants of the Karlsruhe Metabolomics and Nutrition (KarMeN) Study. PloS One (2017) 12(8):e0183228. 10.1371/journal.pone.0183228 28813537 PMC5558977

[73] Darst BF Koscik RL Hogan KJ Johnson SC Engelman CD. Longitudinal Plasma Metabolomics of Aging and Sex. Aging (Albany NY) (2019) 11(4):1262–82. 10.18632/aging.101837 PMC6402508 30799310

[74] Krumsiek J Mittelstrass K Do KT Stuckler F Ried J Adamski J. Gender-Specific Pathway Differences in the Human Serum Metabolome. Metabolomics (2015) 11(6):1815–33. 10.1007/s11306-015-0829-0 PMC4605991 26491425

