# Supplementary material for: Plasma Metabolomics Reveals Dysregulated Metabolic Signatures in HIV-Associated Immune Reconstitution Inflammatory Syndrome
Source: Front Immunol. 2021 Jun 15;12:693074. doi: 10.3389/fimmu.2021.693074 (PMC8239348; doi:10.3389/fimmu.2021.693074)

**Supplementary Figures**

**Supplementary Figure 1.** Changes in CD4^+^ T cell counts (A), plasma HIV-RNA viral load (B), and BMI (C) at the pre-ART, month 1, and month 12 time points for IRIS and non-IRIS groups.

**Supplementary Figure 2.** Identified DEMs for each individual in the IRIS and non-IRIS groups at the pre-ART (A), month 1 (B), and month 12 (C) time points were used in an unsupervised cluster analysis (Ward’s method) of z-score normalized values and dendrograms represent Euclidean distance. In heatmaps, yellow color represents high metabolite levels and blue represents low levels. (D) Based on pathways identified in the co-expressed module analysis comparing IRIS with non-IRIS groups, individual samples including all time points were analyzed using the Gene Set Variation Analysis (GSVA) package and “ssGSE” of the co-expressed pathway modules.

**Supplementary Figure 3.** (A) Heatmap of multi-omics factor analysis (MOFA) analysis showing variance of each latent factor for the combined pre-ART and month 1 time points comparing IRIS and non-IRIS groups. The color scale refers to a discriminant variable measured for each factor. (B-D) List of metabolites, plasma biomarkers, and transcriptomic pathways determining variance for latent factor 1.

**Supplementary Figure 4.** Baseline demographics of study participants stratified by co-infection status is shown in the top panel. Principle component analysis demonstrating the influence of co-infections on the plasma metabolome at baseline in IRIS and non-IRIS groups is illustrated in the bottom panel.

**Supplementary Figure 5.** Differentially expressed metabolites (DEM) were compared among mycobacterial IRIS (orange), other types of IRIS (red), and non-IRIS (purple) groups. Volcano plots depicting the –log(*p*-value) and fold-difference expression of each metabolite and PCA of the identified DEMs comparing mycobacterial IRIS with non-IRIS at the pre-ART time point (A, B), other types of IRIS with non-IRIS at the pre-ART time point (C, D), mycobacterial IRIS with non-IRIS at the month 1 time point (E, F), and mycobacterial IRIS with non-IRIS at the month 1 time point (G, H) are shown. The significance threshold (*p*-value=0.05) is indicated by the red dashed line. Each point represents an identified metabolite with red color indicating significantly upregulated metabolite in the mycobacterial IRIS or other types of IRIS group and blue indicating the opposite.

**Supplementary Figure 6.** Identified DEMs for each patient comparing mycobacterial IRIS and non-IRIS groups at the pre-ART (A), month 1 (B) time points were used in an unsupervised cluster analysis (Ward’s method) of z-score normalized values. Dendrograms represent Euclidean distance. The same analysis was repeated for other types of IRIS and non-IRIS patients at the pre-ART (C) and month 1 (D) time points. In heatmaps, yellow color represents high metabolite levels and blue represents low levels. Based on pathways identified in the co-expressed module analysis comparing different types of IRIS, individual samples at the pre-ART (E) and month 1 (F) time points were analyzed using the Gene Set Variation Analysis (GSVA) package and “ssGSE”.

**Supplementary Figure 7.** All identified metabolites were used in a PCA to visualize the influence of sex on the plasma metabolome in IRIS and non-IRIS groups at the three study time points.

Supplementary Figure 4


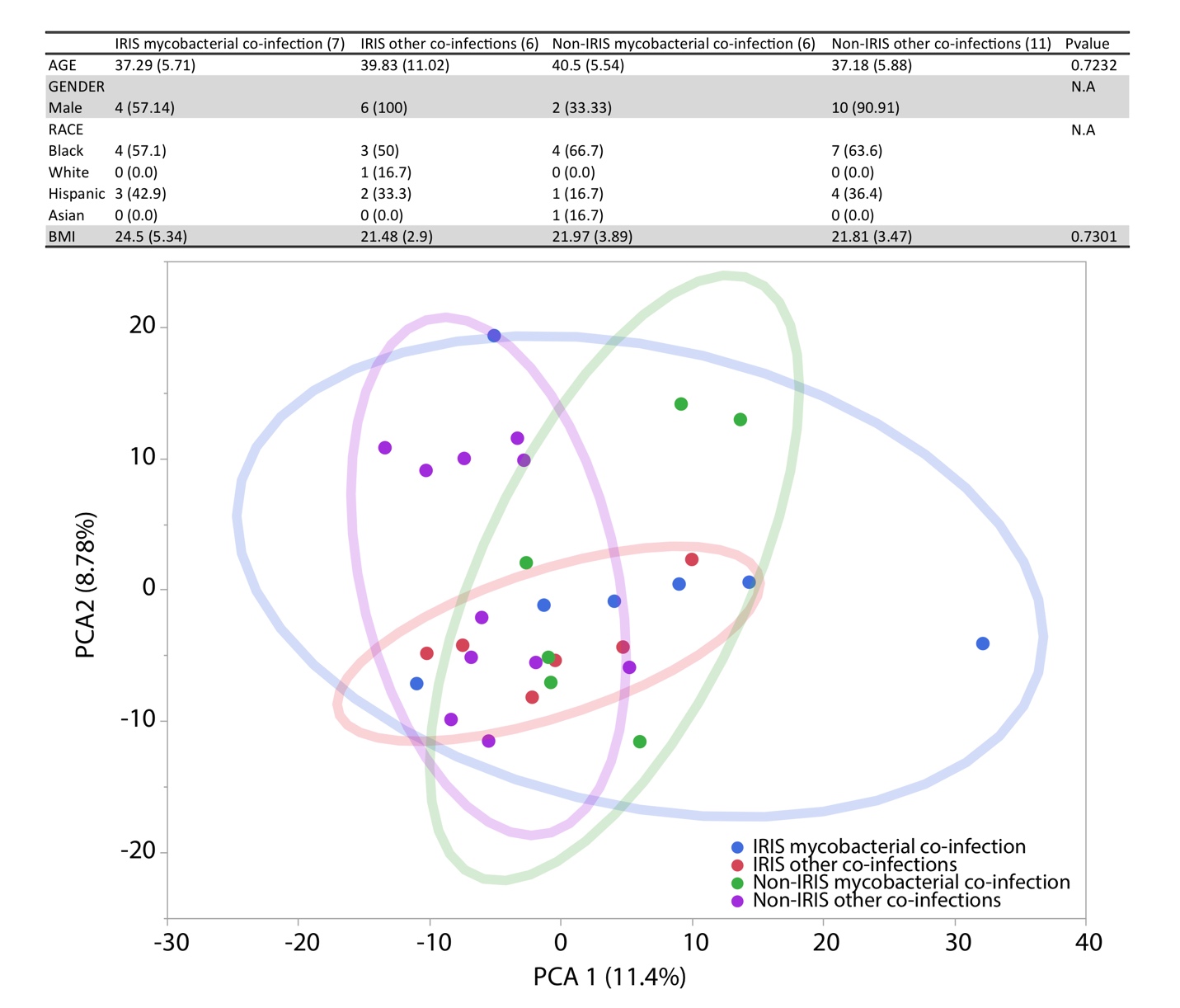


Supplementary Figure 5

Supplementary Figure 6

Supplementary Figure 7


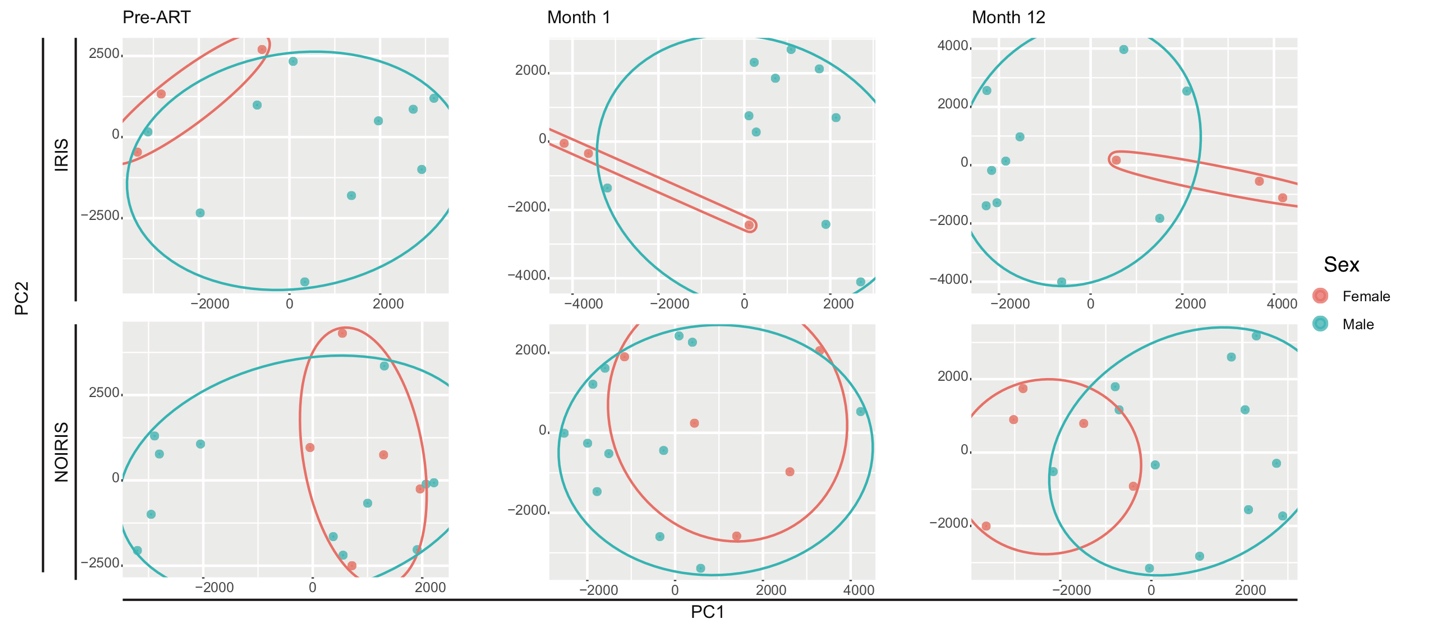

Supplement: Supplementary file 1 [file DataSheet_1.docx]
